# A Case of Facial Paralysis and Lumbago

**Published:** 1881-10-20

**Authors:** C. H. Wagner

**Affiliations:** Waterford, Mississippi


### A CASE OF FACIAL PARALYSIS AND LUMBAGO.

BY C. H. WAGNER, M. D., WATERFORD, MISS.

The motto of this Journal being "Quicquid Præcipies esto Brevis," I will endeavor to relate my case in a concise manner, omitting to state several things which, under other circumstances, I should not have failed to mention.

Eight months ago I was suddenly attacked by paralysis of the muscles of the left side, which are supplied by the portio dura of the 7th pair—æsthesis remaining unimpaired. Besides the usual appearances and symptoms, such as inability to laugh, whistle, raise the eyebrows, shut the eyelids, to spit straight, the chop-fallen cheek, etc., there were others which produced serious misgivings in my mind as to the probable result of the attack, particularly as my father and one of my brothers died of paralysis: they were diplopia, considerable vertigo, a feeling of oppression and heat in the head, buzzing in the ears, and a creeping, gnawing sensation within the lower half of the occiput. The first thing I did was to take a strong drastic cathartic, after the operation of which I immediately commenced the use of the extract of ergot in somewhat larger doses than I should perhaps be willing to administer to others, to relieve the highly probable hyperæmia of the brain and to guard against its possible consequences. Having succeeded in this, I took sulphate of strychnia according to Dr. Hammond's method, but as this article apparently produced no effect, either good or bad, I laid it aside and commenced the use of one-tenth grain of phosphide of zinc and one-twelfth grain arsenite of soda in solution, three times a day alternately. Under the steady use of these remedies, a gradual, progressive improvement took place, which terminated in perfect recovery after a comparatively short time. I will here incidentally mention that the use of tobacco produced a temporary stop in the improvement, so long and as often as it was used.

To my surprise I also got rid, at the same time, of another complaint (and have not felt it since) from which I had been almost constantly suffering, more or less, for the last twenty years—lumbago. As I had then a patient, in the person of a lady 75 years of age, who was constantly afflicted with this pest, and whom I had been treating with every article I had ever used or seen recommended for chronic rheumatism without benefit, I determined to administer these remedies to her, and it gives me pleasure to state that they also cured her. I have used the term "chronic rheumatism," but this is, in my opinion, a misnomer for the affliction, as this complaint has nothing in common with acute rheumatism except pain (on motion), nor does it appear amenable in any degree to the successful remedies employed in that disease, it appearing to be really a simple myodynia—a myopathia of neuralgic character and origin.

